# Shoot transcriptome of the giant reed, *Arundo donax*

**DOI:** 10.1016/j.dib.2014.12.007

**Published:** 2015-01-22

**Authors:** Roberto A. Barrero, Felix D. Guerrero, Paula Moolhuijzen, John A. Goolsby, Jason Tidwell, Stanley E. Bellgard, Matthew I. Bellgard

**Affiliations:** a Centre for Comparative Genomics, Murdoch University, Murdoch, Australia; b United States Department of Agriculture, Agricultural Research Service, Knipling-Bushland U. S. Livestock Insects Research Laboratory, Kerrville, USA; c United States Department of Agriculture, Agricultural Research Service, Cattle Fever Tick Research Laboratory, Edinburg, USA; d Landcare Research Ltd, Private Bag 92170, Auckland 1142, New Zealand

**Keywords:** *Arundo donax*, Giant reed, Shoot, Transcriptome, RNA *de novo* assembly, RNA-Seq

## Abstract

The giant reed, *Arundo donax*, is a perennial grass species that has become an invasive plant in many countries. Expansive stands of *A. donax* have significant negative impacts on available water resources and efforts are underway to identify biological control agents against this species. The giant reed grows under adverse environmental conditions, displaying insensitivity to drought stress, flooding, heavy metals, salinity and herbaceous competition, thus hampering control programs. To establish a foundational molecular dataset, we used an llumina Hi-Seq protocol to sequence the transcriptome of actively growing shoots from an invasive genotype collected along the Rio Grande River, bordering Texas and Mexico. We report the assembly of 27,491 high confidence transcripts (≥200 bp) with at least 70% coverage of known genes in other Poaceae species. Of these 13,080 (47.58%), 6165 (22.43%) and 8246 (30.0%) transcripts have sequence similarity to known, domain-containing and conserved hypothetical proteins, respectively. We also report 75,590 low confidence transcripts supported by both trans-ABBySS and Velvet-Oases *de novo* assembly pipelines. Within the low confidence subset of transcripts we identified partial hits to known (19,021; 25.16%), domain-containing (7093; 9.38%) and conserved hypothetical (16,647; 22.02%) proteins. Additionally 32,829 (43.43%) transcripts encode putative hypothetical proteins unique to *A. donax*. Functional annotation resulted in 5,550 and 6,070 transcripts with assigned Gene Ontology and KEGG pathway information, respectively. The most abundant KEGG pathways are spliceosome, ribosome, ubiquitin mediated proteolysis, plant–pathogen interaction, RNA degradation and oxidative phosphorylation metabolic pathway. Furthermore, we also found 12, 9, and 4 transcripts annotated as stress-related, heat stress, and water stress proteins, respectively. We envisage that these resources will promote and facilitate studies of the abiotic stress capabilities of this exotic plant species, which facilitates its invasive capacity.

## Specifications table

**Table:** 

Subject area	*Biology*
More specific subject area	*RNA-seq transcriptome data of Arundo donax*
Type of data	*Table, figure*
How data was acquired	*2×100 HiSeq (single lane of 100 bases pair-end approach)*
Data format	*Raw FASTQ and processed FASTA*
Experimental factors	*10 g of actively growing shoot, excised approximately 20 cm above soil level*
Experimental features	*Assembled transcriptome of actively growing shoot tissue excised from A. donax grown in field plots*
Data source location	*Laredo, TX, USA*
Data accessibility	*Data is with this article and also available at http://www.ncbi.nlm.nih.gov/GBRH01000000*
	*The assembled and annotated A. donax USA genotype Rio Grande RNA transcriptome has been deposited at DDBJ/EMBL/GenBank under the project accession PRJNA256910*

## Value of data

• First transcriptome sequence data made available in GenBank/DDBJ/Embbl for the *A. donax* invasive Rio Grande basin genotype.
• The *A. donax* shoot transcriptome dataset provides insights into one of the fastest growing terrestrial plants [1].
• *A. donax* has high tolerance to abiotic stresses and its high invasive nature threatens many natural environments and ecosystems.
• The abundant biomass of *A. donax* plants makes it an ideal candidate for biofuel programs [2].

## 1. Experimental design, materials and methods

### 1.1 Plant tissue

Approximately 10 g of *A. donax* shoot tissue was excised from an actively growing shoot, approximately 20 cm above the soil surface of a field plot at the Cattle Fever Tick Research Laboratory, Edinburg, TX, USA. The plants were propagated from plants collected at Laredo, TX in 2008 and designated the Invasive Rio Grande Basin genotype. Excised shoot tissue was taken under natural non-stressed growth conditions and quickly transferred to small vials and placed in dry ice and maintained frozen at −80 °C until transferred into liquid N_2_ during the RNA purification steps.

### 1.2. RNA isolation

Shoot tissue was transferred from storage at −80 °C into liquid N_2_, pulverized, and RNA extracted using the ToTALLY RNA extraction kit according to manufacturer instructions (Life Technologies, Grand Island, NY, USA). A Polytron (Kinematica, Luzern, Switzerland) was used to grind the pulverized tissue for 30 s on ice in the presence of 50 ml of the kit׳s Denaturation Buffer. Following the LiCl precipitation step, a yield of 4 mg of total RNA was obtained. Any traces of contaminating DNA were removed by treatment with TURBO DNA-*free* kit according to manufacturer׳s instructions (Life Technologies) in RNA aliquots of 10 μg. RNA quality was assessed by agarose gel electrophoresis followed by staining with Gelstar Nucleic Acid Stain (Lonza, Rockland, ME) to help verify genomic DNA contamination was not present.

### 1.3. Sequencing and bioinformatics

Sequencing was performed at National Center for Genome Resources (Santa Fe, NM, USA) using the standard Illumina RNA library preparation protocol and a single lane of the HiSeq 100 bases pair-end approach. A total of 181,972,782 pair-end Illumina raw reads were produced, and quality assessed using FASTQC version 0.10.1 [http://www.bioinformatics.babraham.ac.uk/projects/fastqc]. The first 12 bases of all reads were trimmed using seqqtk version 4.19 [https://github.com/lh3/seqtk] to remove sequencing biases. Contigs were *de novo* assembled with trans-ABySS version 1.4.8 [3] and Velvet-Oases version 0.2.08 [4] using *kmer* sizes of 49, 53, 59 and 63. This yielded 368,848 and 1,477,609 transcripts (≥200 bp) produced by trans-ABBySS and Velvet-Oases, respectively. Trans-ABBySS assembled transcripts were further merged using Cap3 [8] at 99.9% sequence overlap identity resulting in 43,822 merged contigs, and 249,590 unmerged transcripts. Velvet-Oases has been shown to produce overall longer assembled transcripts as compared to other assemblers [5,6]. We also found that Velvet-Oases can produce spurious isoforms and these can be removed by selecting representative transcripts for each locus [7].

**Fig. 1:**
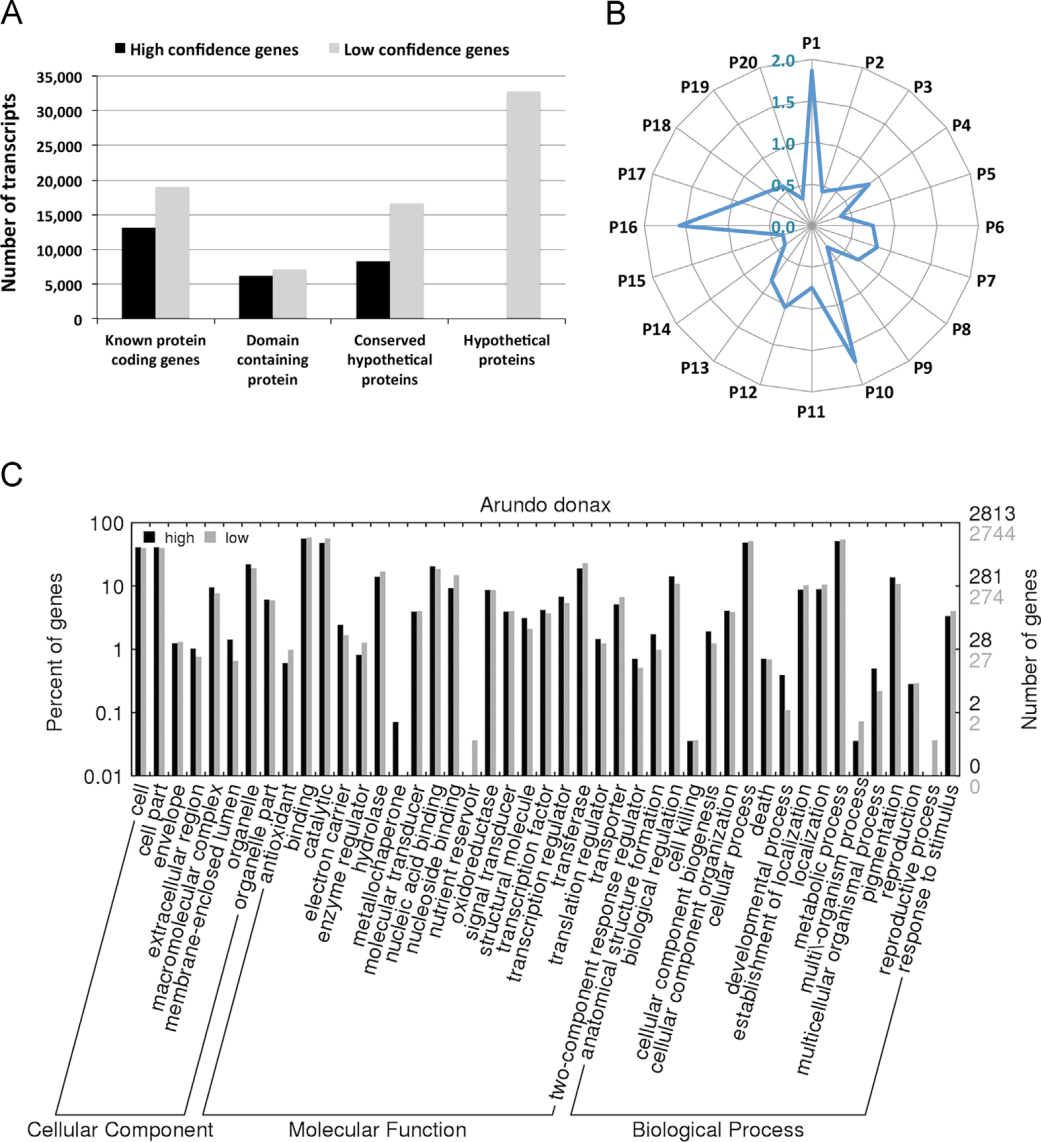
Functional annotation of *A. donax* transcripts: (A) classification of high confidence and low confidence transcripts based on comparison against NCBI NR database. (B) The fold abundance of top 20 KEGG pathways in high confidence transcripts as compared to the low confidence subset is shown. P1=Ribosome; P2=Spliceosome; P3=Ubiquitin mediated proteolysis; P4=Metabolic pathways Oxidative phosphorylation; P5=Plant–pathogen interaction; P6=Proteasome; P7=Protein export; P8=Metabolic pathways, Purine metabolism, Pyrimidine metabolism, RNA polymerase; P9=RNA degradation; P10=Basal transcription factors; P11=Endocytosis; P12=Metabolic pathways, Starch and sucrose metabolism; P13=Peroxisome; P14=Metabolic pathways, N-Glycan biosynthesis; P15=Aminoacyl-tRNA biosynthesis; P16=Natural killer cell mediated cytotoxicity; P17=Base excision repair; P18=Regulation of autophagy; P19=Metabolic pathways, Pyrimidine metabolism; P20=Metabolic pathways, Porphyrin and chlorophyll metabolism. (C) Gene Ontology terms for biological process, molecular function, and cellular componentry were assigned using AutoFACT [9] and summarized using WEGO [10].

We screened assembled transcripts against Poaceae proteins (NCBI NR) and defined as ‘high confidence genes’ those transcripts with sequence identity ≥30% and coverage ≥70% of a known *Poaceae* genes. We also classified as ‘low confidence genes’ those transcripts with partial or no hits to known Poaceae genes that have been assembled by both trans-ABBySS and Velvet-Oases pipelines with 100% sequence identity and reciprocal transcript coverage greater than 90%. We report a total of 103,081 *A. donax* transcripts, of these 27,491 and 75,590 are high and low confidence genes, respectively (Table 1 and Fig. 1A). More than 70% of the high confidence genes were functionally annotated, while only 34.55% of the low confidence genes had partial hits to known and domain-containing *Poaceae* genes (Fig. 1A). We used AutoFACT version 3.4 [9] to functionally annotate transcripts (Supplementary files 1 and 2). The relative abundance of the top 20 KEGG pathways of high confidence genes as compared to the low confidence gene set is shown in Fig. 1B. We found 1.86, 1.71 and 1.58 fold increase of the number of genes assigned to the spliceosome, metabolic pathways of purine metabolism and peroxisome among high confidence genes (Fig. 1B). Fig. 1C shows the top Gene Ontology annotations found among high and low confidence genes. Interestingly, two genes with copper ion binding and transport function were only found among the high confidence genes, while genes involved in nutrient reservoir activity and reproductive growth were only found among the low confidence genes (Fig. 1C). The resources generated in this study will facilitate comparative transcriptomics analyses of invasive plant species.

## 2. Direct link to deposited data

Deposited data can be found here: http://www.ncbi.nlm.nih.gov/GBRH01000000.

## 3. Nucleotide sequence accession number

The assembled and annotated *A. donax* USA genotype Rio Grande RNA transcriptome has been deposited at DDBJ/EMBL/GenBank under the project accession PRJNA256910. This Transcriptome Shotgun Assembly project has been deposited at DDBJ/EMBL/GenBank under the accession GBRH00000000. The version described in this paper is the first version, GBRH01000000.

## Conflict of interest

The authors declare that there is no conflict of interest on any work published in this paper.

## Figures and Tables

**Table 1:** *Arundo donax* transcriptome assembly statistics.

	**High confidence genes**	**Low confidence genes**
Number of transcripts	27,491	75,590
Total size of transcripts	32,326,850	55,020,434
Longest transcript	14,995	8091
Shortest transcript	200	200
Number of transcripts>1K nt	13,877 (50.5%)	14,879 (19.7%)
Number of transcripts>10K nt	2 (0.0%)	0 (0.0%)
Number of transcripts>100K nt	0 (0.0%)	0 (0.0%)
Mean transcript size	1176	728
Median transcript size	1008	584
N50 transcript length	1413	870
L50 transcript count	7811	19,821
Transcript %A	24.16	26.16
Transcript %C	25.11	23.28
Transcript %G	26.36	24.02
Transcript %T	24.37	26.53
Transcript %N	0	0

## Supplementary materials

Supplementary file 1. Functional annotation of *Arundo donax* high confidence genes.

Supplementary file 2. Functional annotation of *Arundo donax* low confidence genes.
